# Improvement of stuttering after administration of methylphenidate - a case report

**DOI:** 10.1016/j.pmip.2022.100092

**Published:** 2022-02-09

**Authors:** Shahriar SheikhBahaei, Mutahir Farhan, Gerald A. Maguire

**Affiliations:** a Neuron-Glia Sign aling and Circuits Unit, National Institute of Neurological Disorders and Stroke (NINDS), National Institutes of Health (NIH), Bethesda, MD 20892, USA; b Department of Psychiatry and Neuroscience, University of California, Riverside School of Medicine (UCR SOM), Riverside, CA 92521, USA; c iStutter/Doc1 Health – California University of Science and Medicine, Newport Beach, CA 92660, USA

**Keywords:** Childhood-onset fluency disorder, Stuttering, Dopamine, Methylphenidate, Personalized medicine

## Introduction

Stuttering, also known as childhood-onset fluency disorder, is a condition associated with disruptions in the timing and initiation of speech with common symptoms of repetition, pausing, and/or prolongation of words. Stuttering can be associated with motor or phonic tics, cognitive avoidance, and social anxiety [1,2]. There is also a strong comorbidity with stuttering and attention-deficit/hyperactivity disorder (ADHD) [3]. By definition, this disorder may interfere with an individual’s social, occupational, or academic achievement. Stuttering most commonly presents in early childhood and affects approximately 4% of children. Although spontaneous remission may occur, stuttering persists in greater than 1% of the adult population [4]. Significant empirical pharmacologic and basic science data reveal dopamine hyperactivity to be common among individuals who stutter. With such, stimulant medications acting upon dopamine, for the use of comorbid ADHD, often worsen stuttering [5,6]. However, we report a case where the utilization of a stimulant medication, methylphenidate, was associated with an improvement of the patient’s stuttering.

## Case

A 28-year-old Asian-American male was evaluated in the psychiatry outpatient setting for pharmacologic treatment of his ADHD. In addition to his ADHD, the patient also carried the diagnosis of Childhood-Onset Fluency Disorder (Stuttering). At the time of his presentation, the patient was not taking any medication for his stuttering or ADHD. The patient’s stuttering was developmental in nature with onset in early childhood and persistence into adulthood. The patient ‘s prenatal course, birth and developmental history were all unremarkable. The patient has no family history of stuttering. At the time of the initial visit, the patient was a graduate student and relayed several stressors related to his ability to focus on his studies. The patient ‘s stuttering was negatively impacting his social communication, quality of life and academic performance.

The patient had been diagnosed with ADHD 14 years prior by a neuropsychologist (testing records no longer available) and the subject did not receive pharmacologic treatment. In regard to his stuttering the patient did have numerous courses of speech therapy throughout childhood, adolescence and young adulthood with a lack of response. Upon presentation to our care, the subject ‘s baseline, pre-treatment Subjective Screening of Stuttering (SSS) [7] revealed a total score of 115. Initially, the patient was begun on atomoxetine, titrated to 80 mg per day, for the treatment of his ADHD given the agent’s lack of action upon the dopamine system. He exhibited a mild response in regard to his ADHD with no worsening of his stuttering. A course was then begun to address his stuttering with the use of dopamine 2 (D2) receptor active agents, lurasidone up to 60 mg per day and aripiprazole up to 15 mg per day, without efficacy. Given the patient ‘s mild response of his ADHD with atomoxetine, he requested a course of an agent that may yield a greater response. The patient was discontinued from the atomoxetine and began a low dose of methylphenidate [a dopamine transporter (DAT) blocker [8]], 5 mg twice a day, which resulted in significant improvement in his attention and focus, and unexpectedly, also resulted in improvement in his stuttering. The patient subjectively noted feeling calmer and less anxious with his speech. His SSS after being treated with methylphenidate for six weeks revealed a score of 74, noting a 36% improvement in his stuttering.

## Discussion

Various etiologies of stuttering have been described including genetic and autoimmune but other causes are likely yet to be characterized [9,10]. Abnormal dopamine activity in the basal ganglia has been shown to be strongly associated with stuttering [11], and it is hypothesized that an imbalance in dopamine activity may be linked to stuttering [12,13]. This hypothesis is also supported by several case reports and studies discussing the use of methylphenidate for patients with ADHD [8,14–16]. It is reported that the use of methylphenidate successfully reduced stuttering in an 18-year-old patient [15]. Moreover, a randomized controlled study evaluating the influence of methylphenidate on frequency of stuttering reported that participants exhibited a decrease in the frequency of stuttering with methylphenidate [14]. In rodent models, methylphenidate was also shown to inhibit dopamine reuptake in the striatum, nucleus accumbens, olfactory tubercle, and prefrontal cortex [16].

Conversely, several case reports have postulated that methylphenidate may play a role in the onset of stuttering [5,6]. A 7-year-old patient with ADHD showed onset of stuttering 10 days after initial treatment with methylphenidate [5]. Similarly, a connection between using methylphenidate to treat ADHD and an acute onset of stuttering in a another 7-year-old patient was reported [6]. Methylphenidate-induced stuttering disappeared with discontinuation of medication, and re-emerged with a subsequent trial of treatment [6].

Dopamine signaling and distribution in neurons and astrocytes are primarily modulated by the DAT, which transport dopamine into presynaptic terminals, and by vesicular monoamine transporter (VMAT-2), which transport dopamine into synaptic vesicles [17–19]. Each dopaminergic neuron has a basal activity, and perturbation of either DAT or VMAT-2 alters extracellular and intracellular dopamine concentrations, respectively. In addition, the basal dopamine activity likely determines the level of methylphenidate-induced increases in dopamine in subjects [20].

Due to the conflicting nature of studies showing stuttering being induced by or improved with methylphenidate, it may be appropriate to hypothesize that dopamine serves a complicated and variable role in stuttering. It has been proposed that certain patients who responded well to dopamine stimulants and poorly to D2-antagonist agents may have a different subtype of stuttering compared to those who responded well to D2-antagonist agents and poorly to dopamine stimulants [12]. It is accepted that methylphenidate works by binding to the DAT [8] in presynaptic cell membranes to block the reuptake of dopamine. The findings that methylphenidate can attenuate stuttering in a subgroup of people who stutter supports that stuttering might not simply be a result of excess dopamine, but rather related to a misbalance between activation of D1 and D2 receptors. In addition, methylphenidate via altering VMAT-2 function, may redistribute dopamine vesicles from the plasmalemmal membrane-associated into cytoplasmic pools (see [21,22] for more information). Therefore, the therapeutic effects of methylphenidate may occur when it elicits slow, steady-state synaptic dopamine release [20]. In addition, recently it was proposed that astrocytes, the star-shaped non-neuronal cells in the brain, might have a key role in pathophysiology of stuttering [18,23,24]. The involvement of astrocytes in dopaminergic signaling and their modulatory role of neuronal circuits in the basal ganglia [18] further suggest that the underlying mechanisms of stuttering are not simple.

In our patient, we observed a 36% improvement in his stuttering (measured by SSS) six weeks after taking methylphenidate. SSS is designed based on measure the self-reports scales of people who stutter before, during, and after their treatment [7]. To do so, SSS screens three areas of stuttering severity, level of control, and avoidance. Each of these areas has two to three elements which are assessed with a one-to-nine rating scale, with a higher SSS number corelates with a more severe stuttering in speech.

Due to the variability in stuttering presentations and responses to dopamine agents, our report suggests that different underlying mechanisms in the dopaminergic circuits may lead to stuttering. Given the variability in observed published cases, one can conclude that a simple increase or decrease in activities of D1 or D2 receptors will not translate simply to a uniform response across individuals who stutter. Despite some evidence where D2 antagonists improve stuttering and stimulant agents, which increase dopamine activity, worsening stuttering, our report further supports the hypothesis that stuttering may be a heterogenous disorder requiring personalized therapies tailored to each individual.

## Abbreviations:

ADHD: Attention-deficit/hyperactivity disorder
DAT: dopamine transporter
SSS: Subjective Screening of Stuttering
VMAT-2: vesicular monoamine transporter 2
